# The role of baseline serum 25(OH)D concentration for a potential personalized vitamin D supplementation

**DOI:** 10.1038/s41430-022-01159-6

**Published:** 2022-05-23

**Authors:** Paola G. Ferrario, Bernhard Watzl, Christian Ritz

**Affiliations:** 1 Department of Physiology and Biochemistry of Nutrition, Max Rubner-Institut, Karlsruhe, Germany; 2 National Institute of Public Health, University of Southern Denmark, Copenhagen, Denmark

**Keywords:** Biomarkers, Computational biology and bioinformatics

## Abstract

There are many examples in clinical nutrition with individuals responding differently to a dietary intervention. Here, personalized nutritional recommendations could be targeted, tailoring interventions to address plurality in response. For this, we reconsidered two randomized controlled trials investigating whether two different supplementation doses of cholecalciferol resulted in an increased serum 25(OH)D concentration. Three different statistical methods were applied in order to identify participants who would enjoy an increased benefit from receiving a specific dose of cholecalciferol. We concluded that, for individuals with high baseline serum 25(OH)D concentrations, only the high supplementation dose will lead to a substantial increase in the serum 25(OH)D concentration. The presented statistical methods can be adequately used for more individualized approaches.

## Introduction

Dietary interventions may easily lead to differential responses. In fact, a great heterogeneity in absorption, metabolism and excretion of nutrients [1] and in response to different dietary interventions has already been established and largely documented in the literature. To give just a few examples, heterogeneity in weight loss [2], in postprandial glycaemia [3], or in stimulation after caffeine intake [4] were observed. Specifically, the efficacy of a dietary intervention may vary from participant to participant. In fact, many characteristics of the individual participants may play a decisive role in their response to a dietary intervention. Such characteristics can be tentatively summarized in so called “metabotypes” [5], defined as individual metabolic states reflecting what has been encoded by the genome, and modified by diet, environmental factors, and the gut microbiome, as quantifiable readouts of the biochemical state, that can span from normal physiology to diverse pathophysiologies.

The standard statistical analyses of randomized trials planned for comparing different dietary interventions typically evaluate the average effect, i.e., an average effect calculated for the study population receiving a certain dietary intervention. In consequence, by taking the average, a possible outcome of such analyses may be that no differences are found between two interventions. However, even if no differences on average could be established, a great variability between participant responses to interventions may still be present, but this often remains unexplored (see reference [1 and 2] and [2], for instance). Are we satisfied with the statement that there are no differences on average between these interventions even though some individuals could benefit more than others? Conversely: can a dietary recommendation be really given, which is deemed to be effective on average, if it is clear that a subgroup of individuals will not benefit from it at all? The answer to both questions is no. In fact, the field of personalized nutrition has been put forward for addressing this type of questions. Specifically, one of the aims of personalized nutrition is to address plurality in intervention responses using individualized information, to deliver nutritional recommendations that are expected to be more suitable and effective than generic recommendations [6]. Related to the concept of personalized nutrition, the concept of precision nutrition is likewise widely used in the literature, but to date there is no universal consensus on the definitions of these terms. In practice, we will use the abbreviation PN for both terms and assume the definitions given by Ordovas et al. [7] throughout this article. Two large studies conducted in a PN context are noteworthy, namely the Food4Me [8] and the PREDICT 1 [3] studies within the PREDICT program.

One further path of PN is represented by the so called outcome-based approaches as described in Ferrario et al. [9]. Here, the initial step consists of re-using existing data documenting heterogeneity in response to interventions, while the aim is to achieve an improvement in a pre-specified outcome by preferring one dietary intervention over others, according to some specific individual characteristics of individuals. Since individualized information is involved in order to ultimately be able to provide differential nutritional recommendations, these approaches could be used to develop criteria for a potential personalized nutrition. In this paper, we are going to show how outcome-based approaches can provide insights regarding differential individual nutritional benefits. We will use vitamin D supplementation as example.

Specifically, we revisited a completed randomized controlled trial (RCT) covering vitamin D supplementation that explored the safety and efficacy of supplementation with two different doses of cholecalciferol [10]. Supplementation by the two doses was found to be effective on average over time; however, this does not mean that they were equally beneficial for all participants. In fact, the high variance in the serum vitamin D (25(OH)D) levels, especially after the intervention with the higher dose, suggests that some participants may benefit more and others less.

To scrutinize this assumption, i.e., in order to identify participants who would benefit more from a specific supplementation with vitamin D, we employed three statistical methods: an initial classical subgroup analysis, similar to that often used in medical research [11], a method based on interaction terms with a quantitative baseline covariates, previously applied to weight loss studies (Ritz et al. [2] and Hjorth et al. [12]), and a more general method allowing for nonlinearity in the covariates, originally developed in the context of personalized medicine (Matsouaka et al. [13]).

## Methods

### Reconsidering data from a randomized controlled trial (RCT)

We reconsidered the study by Steenhoff et al. [10], a RCT carried out in 2012 in Gaborone, Botswana. The goal of this study was to investigate whether two different supplementation doses of cholecalciferol (4000 IU/day vs. 7000 IU/day) would safely result in an increased serum 25(OH)D concentration. Serum 25(OH)D concentrations were measured at baseline, after 6 and after 12 weeks of intervention. Specifically, this RCT was designed as pilot, double blind study, with randomized parallel assignment to two possible interventions (i.e., as between-groups design) and in conjunction with repeated measurements at three time points (also known as within-subject design). Moreover, the study examined other parameters linked to the intervention (like HIV-specific anti-retroviral therapy since the recruited patients had HIV), but these are not the focus of this work.

### Statistical analysis

#### Preliminary analysis: subgroup analysis and linear mixed models

Baseline characteristics of the participants and serum 25(OH)D concentrations were summarized using means ± standard deviations. 60 children and adults living in Botswana were included, aged between 5.0 and 50.9 years (with average age of 19.5 ± 11.8, 50% male) [10]. 30 participants orally consumed 4000 IU/day of cholecalciferol for twelve weeks as standard intervention, and 30 participants took 7000 IU/day, as experimental intervention (thereof 29 completed the trial).

To investigate the heterogeneity in the response of the participants to the intervention, a subgroup analysis was carried out, estimating intervention effects within subgroups [11]. Specifically, the subgroups of participants were determined according to their baseline serum 25(OH)D concentrations: a post-hoc cut-off for serum 25(OH)D 30 ng/ml was used, resulting in two subgroups. In fact, the value of 30 ng/ml was chosen as the minimum serum 25(OH)D concentration for serum sufficiency, as documented in Maretzke et al. [14]. Subsequently, two separate regression models for the two subgroups were fitted to assess intervention effects for participants starting or not with serum 25(OH)D sufficiency. Significance level was set at 0.05/2 = 0.025, using a Bonferroni correction that reflected the number of subgroups.

To investigate the intervention effect and change over time in serum 25(OH)D concentration, a linear mixed effect model for serum 25(OH)D concentration was fitted depending on dose, time, and baseline 25(OH)D concentration as a covariate. Moreover, the models also included relevant covariate adjustments: age, sex as fixed effects and participant-specific random intercept effects. Most importantly, models included also body weight as an additional covariate; in fact, serum 25(OH)D concentration is known to vary conditionally to total body fat mass and to be poor in obese individuals, as discussed by [15].

#### Parametric prediction models for estimation of individualized intervention effects

To further investigate intervention outcome by the two doses, predictions of the change in serum 25(OH)D concentration after intervention (i.e., of the difference between the serum 25(OH)D concentration at week 12 and at week 0) were generated conditional to dose, time, baseline separated for low and high supplementation dose.

Furthermore, to investigate the intervention outcome by the two doses and among different levels of baseline variables, i.e., to assess a possible *modulation* of the supplementation effect by baseline characteristics, we considered linear mixed models with baseline covariate-intervention interaction terms.

Specifically, a linear mixed model for the serum 25(OH)D concentration was fitted considering (i) participant-specific random effects (ii) baseline serum 25(OH)D concentration (iii) interaction term between baseline serum 25(OH)D concentration and a categorial variable combining the two time points (after 6 and after 12 weeks intervention) and the two supplementation doses iv) sex, age, and body weight as adjusting covariates.

Using mathematical notation, we started by general multiple linear regression models for the serum 25(OH)D concentration in the two intervention groups, k = 1 or =2, (k = 1 standard intervention, k = 2 experimental intervention, as defined in *Preliminary analysis: subgroup analysis and linear mixed models*)

1 $$Y_k = X_k\beta _k + \varepsilon _k = m_k\left( x \right) + \varepsilon _k,k = 1 \vee 2$$

Then, the focus was on the difference

2 $$m_2( {x,\hat \beta _2} ) - m_1( {x,\hat \beta _1} )$$

where $m_1( {X,\hat \beta _1} )$ and $m_2( {X,\hat \beta _2} )$were the two multiple linear regressions within each intervention group, *k* = 1v2, respectively, with parameter estimates $\hat \beta _1$ and $\hat \beta _2$.

Specifically, the difference between the estimated parameters representing the model intercepts by the two supplementation doses (at week 12) were considered. Moreover, the difference between the estimated coefficients representing the model slopes by the two supplementations (again at week 12) were considered. The sum of these differences quantified the average net gain in the 25(OH)D concentration for preferring one dose over the other. Finally, confidence intervals were constructed for the predicted values via the delta-method.

A further, analogous linear mixed model was fitted conditionally to both baseline 25(OH)D concentration and age (here, considering one more slope term), generalizing the method previously described in [12] and [2].

#### Semi-parametric prediction models for estimation of individualized intervention effects

Linear models, as the ones described in *Semi-parametric prediction models for estimation of individualized intervention effects*, are on one hand “simplifying”, but at the expense of imposing the assumption of a “linear shape”. To overcome this strong assumption, nonparametric approaches can be adopted, in order to achieve flexibility in the estimation.

Following a two-step procedure as in Matsouaka et al. [13], we added a non-parametric step to the fitted parametric regression line described in *Parametric prediction models for estimation of individualized intervention effects*. This results in a semi-parametric, robust approach accounting for possible model misspecification by the parametric step.

Specifically, the non-parametric step consisted of an estimation of the parameters obtained by the parametric fit with the help of different local averaging techniques. This localization is achieved via a weighting function or general kernel *k*(*t*_0_, *t*_1_), which assigns a weight to *t*_1_ based on its distance from *t*_0_, depending on the bandwidth *h*_*n*_, giving the width of the neighborhood.

We adopted and compared kernel weights and local regression approaches (here with bandwidth as smoothing parameter *h*_*n*_ set to 0.6 or to 0.8). In this way, the net gain in the 25(OH)D concentration for preferring one dose over the other was estimated semi-parametrically. This means that each regression function in [2] were estimated by

$$m_k\left( x \right) = {\sum\limits_{i = 1}^nW_i\left( x \right)Y}$$

for *k* = 1v2, with following weights *W*_*i*_

$$W_i\left( {x,X_1, \ldots ,X_n} \right) = \frac{{k\left( {\frac{{x - X_i}}{{h_n}}} \right)}}{{\mathop {\sum }\nolimits_{i = 1}^n k\left( {\frac{{x - X_i}}{{h_n}}} \right)}}$$

where *k*() may be the Nadaraya-Watson Kernel [13].

## Results

### Comparing intervention effects and heterogeneity in response

The individuals were 50% male, aged 19.5 ± 11.8 years and weighing 58.3 ± 13.9 kg. The intervention by the two doses of cholecalciferol resulted in a change in the serum 25(OH)D concentration. Specifically, the serum 25(OH)D concentrations (in ng/ml) were 36.5 ± 9.3 before and 54.8 ± 13.0 after the standard intervention. For the experimental intervention, serum 25(OH)D values were 34.5 ± 9.5 before and 56.5 ± 22.4 after the twelve weeks. With the fitted linear mixed effect model for serum 25(OH)D concentration as described in *Preliminary analysis: subgroup analysis and linear mixed models*, no significant difference between the two supplementation doses could be shown (estimated difference equal to 5.29 ng/ml [95% CI: −2.09, −12.67]; *p* = 0.175), i.e., no difference in average could be found on induced change in the level of serum 25(OH)D after supplementation by 4000 IU/day or 7000 IU/day, having included baseline, sex, age, and body weight as covariates. Moreover, we were able to observe an inversely significantly effect of body weight on serum 25(OH)D concentration. Specifically, we obtained an estimated effect equal to −0.45 ng of 25(OH)D/ml per kg body weight ([95% CI: −0.73, −0.17]; *p* = 0.002). In addition, a great variability between participants’ response was observed. Specifically, the difference between the maximum and minimum serum 25(OH)D value was equal to 100 ng/ml at the end of the experimental and equal to 54 ng/ml at the end of the standard invention, respectively, and the standard deviations equal to 22.4 and 13.0, respectively, which gives an indication of heterogeneity in response to the intervention between the participants (compare also the second line in Table 1, Supplementary Materials).

### Stratifying individuals by subgroup analysis

For the participant group with baseline serum concentrations smaller than 30 ng/ml, the estimated difference between the two interventions was equal to −2.23 ([97.5% CI: −22.11, 17.75]; *p* = 0.829), i.e., no statistically significantly difference between the two interventions could be shown. Thus, no resulting difference was observed between the two interventions overall for all participants as well as within this subgroup. On the other hand, i.e., for the group with baseline serum concentrations greater than 30 ng/ml, the estimated difference between the two interventions was equal to 8.46 ([97.5% CI: −0.49, 17.41]; *p* = 0.043) respectively. Hence, no significant differences were observed between the two interventions (after Bonferroni correction). However, the heterogeneity in response remained unexplained and may be further investigated.

### Estimating the net gain in serum concentration for one intervention over the other (parametrically)

**Fig. 1:**
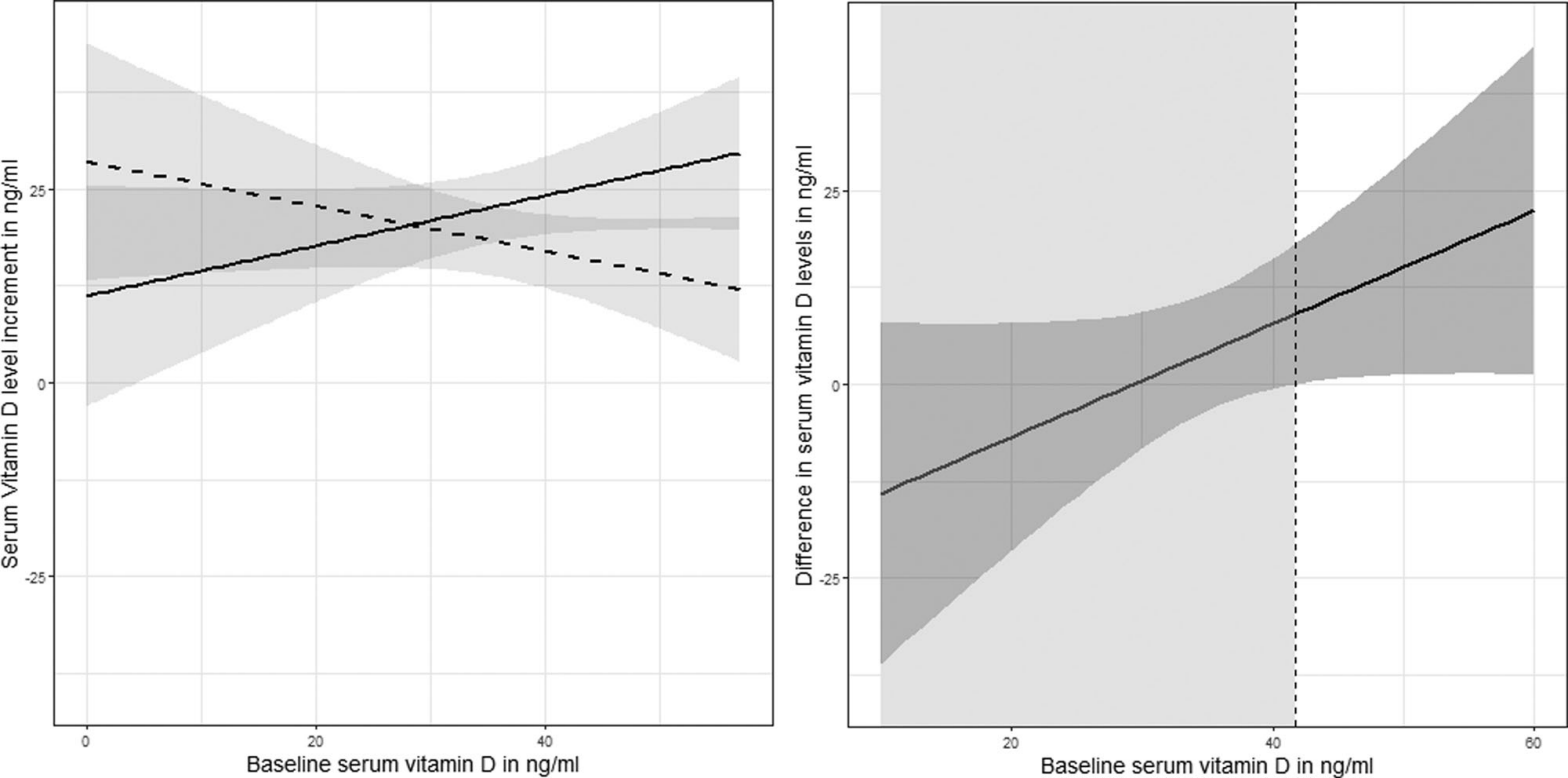
Parametric fits. The dashed, decreasing line visualizes prediction of serum change to baseline after supplementation of 4000 IU/day; the solid, increasing line the prediction of serum change to baseline after supplementation of 7000 IU/day, with marginal confidence bands, respectively. Right-hand side: Fitted regression line with 95% confidence band by the parametric model with interaction term for the serum 25(OH)D concentration conditional to the baseline. White background for confidence band not crossing the zero.

Predictions of the difference between the serum 25(OH)D level at week 12 and at week 0 were generated with respect to the baseline, separated for low and high supplementation doses and visualized at once (Fig. 1, left-hand side).

By the parametric model (as described in *Parametric prediction models for estimation of individualized intervention effects*) a higher effectiveness of supplementation of 7000 IU/day than of 4000 IU/day was predicted for higher baseline serum 25(OH)D concentrations. For instance, for a baseline value equal 40.5 ng/ml the estimated net gain was equal to 8.60 with a 95% confidence interval equal to [0.02, 17.17] (i.e., not containing the zero). This could mean that an individual having a baseline concentration of 40.5 ng/ml needs to receive the higher supplementation of 7000 IU/day to considerably increase the serum 25(OH)D concentration.

The fitted regression line with 95% confidence band for the serum 25(OH)D concentration is visualized with respect to the baseline (Fig. 1, right-hand side): the fitted regression line with confidence band on the white background corresponds to baseline values higher than 40.5 ng/ml. Here, the net gain in serum 25(OH)D concentration by supplementation of 7000 IU/day over supplementation of 4000 IU/day is significantly different from zero and becomes positive. Further details of the estimated parameters and confidence intervals can be found in the Supplementary Materials (Table 2). Furthermore, using the fitted model considering both baseline concentration and age as predictors, higher effectiveness of supplementation of 7000 IU/day than of 4000 IU/day was predicted for higher baseline serum 25(OH)D levels, and this irrespective of individuals’ age. This would mean that knowing the age of a participant would not support the decision of supplementation by the higher dose over the smaller dose. However, since the considered RCT had a restricted number of participants who were relatively young (average age of 19.5), and considering children and adults concomitantly, representativeness of human’s age is lacking and therefore any statement about age as predictor at this point lacks generality.

### Estimating the net gain in serum concentration for one intervention over the other (semi-parametrically)

**Fig. 2:**
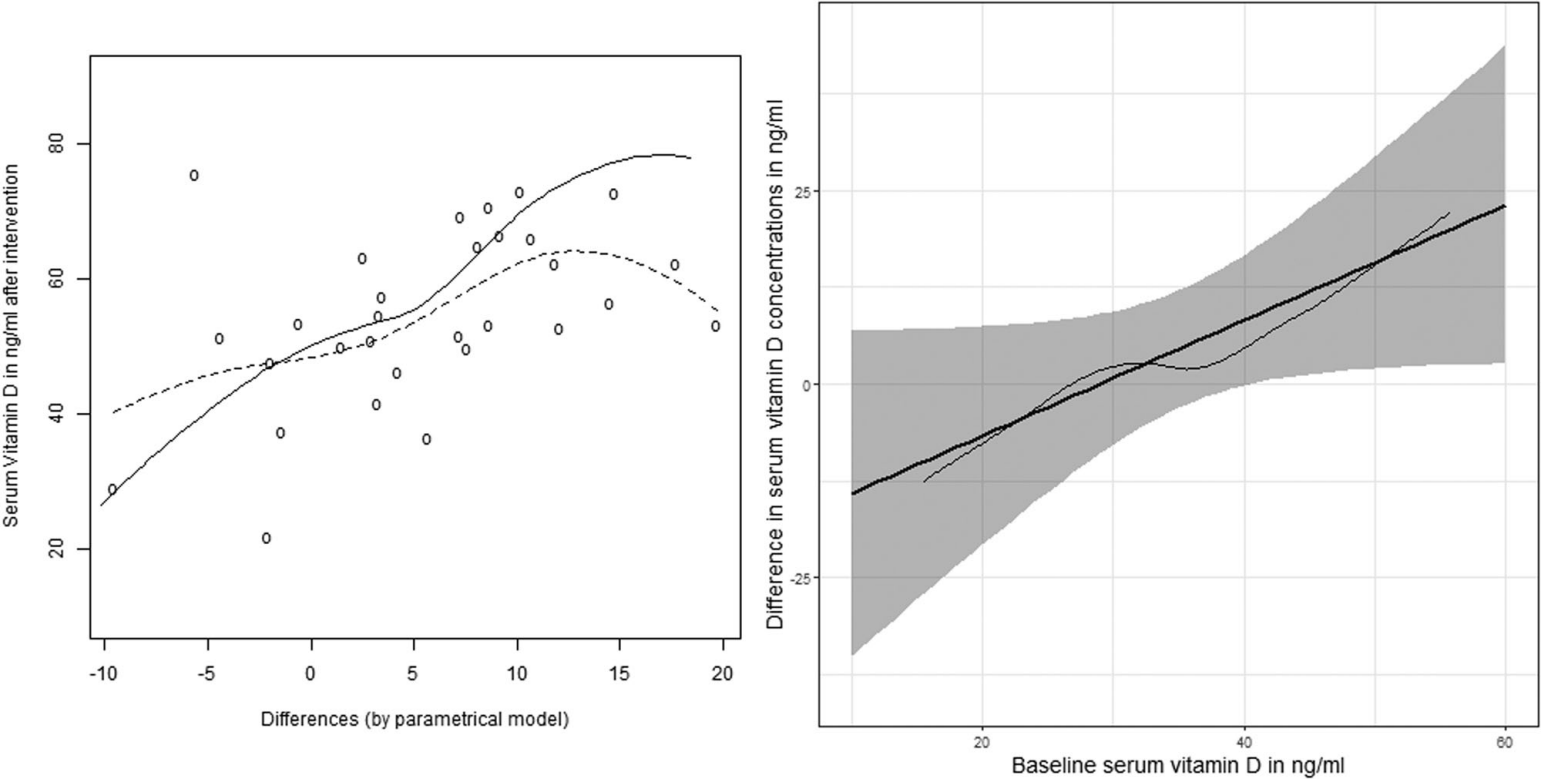
Semi-parametric fits. Left-hand side: Semi-parametric fit conditional to the differences estimated parametrically: dashed line for standard intervention (4000 IU/day); solid line for experimental intervention (7000 IU/day). Right-hand side: Fitted regression line with 95% confidence band by a parametric model (as in Fig. 1) as well as fit by the semiparametric model.

By adding a non-parametric step (as described in *Semi-parametric prediction models for estimation of individualized intervention effects*), prediction in serum 25(OH)D concentration is no longer linear; predicted serum concentration is visualized at week 12 conditional to the differences calculated by the parametric step, separated for low and high supplementation doses; both predictions are visualized simultaneously: the dashed line visualizes semi-parametric prediction of serum 25(OH)D concentration after supplementation of 4000 IU/day; the solid line the semi-parametric prediction of serum 25(OH)D concentration after supplementation of 7000 IU/day respectively (Fig. 2, left-hand side). Moreover, semi-parametric estimation of the net gain in the 25(OH)D concentration for preferring one dose over the other is visualized concomitantly with the linear regression line (with 95% confidence band) by the parametric model (Fig. 2, right-hand side). Qualitatively, it could be said that both estimates, i.e., by imposing the linear form or renouncing to this assumption, give comparable results. Considering the description of outcome-based approaches (Ferrario et al. [9]), the semi-parametric model could be addressed as well as outcome-based approaches, useful in personalized nutrition context.

### Replicability of the findings in a further randomized controlled trial (RCT) as external, independent dataset

In order to check for replicability of the findings, we consider a further, independent dataset: we reconsidered the study by [16], carried out in 2013 in Finland.

### Study design

This study aimed to investigate effects of vitamin D supplementation, by a three-armed trial over 20 weeks. The three intervention groups were: placebo, supplementation of 1600 IU/day vitamin D (which corresponds to 40 µg/day, as given in [16]), and supplementation of 3.200 IU/day (in [16]: 80 µg/day). The design of this study was therefore a purely between-groups design. The focus of this study was on the effect of vitamin D3 supplementation on glucose metabolism. For this, 71 pre-diabetic individuals were recruited, above the age of 60, both male and female, exhibiting evidence of disturbed glucose homeostasis but no type 2 diabetes, and with a body mass index between 25 and 35. An additional inclusion criterion was a baseline serum 25(OH)D concentration of below 30.4 ng/ml (in [16] as 75 nmol/L), measured from venous blood samples.

Unlike in the article [16] where the vitamin D dosage are given in µg/day, we convert them here in IU/day so that they are presented consistently throughout this article (one µg/day corresponds to 40 IU/day). Moreover, unlike in the article [16] where the concentrations are given in nmol/L, we convert them here in ng/ml so that they are presented consistently throughout this article, too (conversion of the nmol/L concentrations in to ng/ml concentrations dividing by 2.5).

### Modified design and statistical analysis

In the present study, we focused on the sub-group of 49 participants of the above-described cohort that received one of the two doses of vitamin D, i.e., we did not consider the 22 participants of the placebo group. Thereby, 25 participants who took orally 1600 IU/day of Vitamin D for five months are considered as receiving the standard intervention, and 24 participants who took 3200 IU/day as receiving the experimental intervention. Moreover, we included only individuals for whom serum 25(OH)D concentrations were available from both the start and the end of the trial.

Also in this dataset, baseline characteristics of the participants and serum 25(OH)D concentrations were summarized using means ± standard deviations. Since only participants with baseline serum 25(OH)D concentration of below 30.4 ng/ml were admitted according to the inclusion criterion, a corresponding subgroup analysis as described in *Preliminary analysis: subgroup analysis and linear mixed models* with a post-hoc cut-off for serum 25(OH)D 30 ng/ml cannot be performed. To investigate the intervention effect and change in serum 25(OH)D concentration, a linear mixed effect model for serum 25(OH)D concentration was fitted depending on dose and baseline as covariate. Moreover, the models also included relevant covariate adjustments: age, sex and BMI (BMI since body weight was not available in this dataset).

To further assess outcome modulation by the two doses and among different levels of baseline variables, we considered prediction models as described in *Parametric prediction models for estimation of individualized intervention effects*, calculating the net gain in the 25(OH)D concentration for preferring one dose over the other and testing for significant difference.

## Results

The individuals were 86% male, aged 66.3 ± 4.9 years and with BMI 29.1 ± 2.9. The intervention by the two doses of vitamin D resulted in a change in the serum 25(OH)D concentration. Specifically, the serum 25(OH)D concentrations 23.6 ± 3.04 before and 34.28 ± 6.24 ng/ml after the standard intervention. For the experimental intervention, serum 25(OH)D values were 23.12 ± 4.12 before and 41.08 ± 9.26 after the 20 weeks. As in [16], we saw a difference in males and females with regard to serum 25(OH)D concentration.

According to the parametric model (as described in *Parametric prediction models for estimation of individualized intervention effects*), higher efficacy of supplementation by 3200 IU/day than by 1600 IU/day was predicted for higher baseline serum 25(OH)D concentrations. For instance, for a baseline value equal 20 ng/ml the estimated net gain was equal to 5.44 with a 95% confidence interval equal to [0.036, 10.836] (i.e., not containing the zero). This could mean that an individual having a baseline concentration of 20 ng/ml needs to receive the higher supplementation of 3200 IU/day to considerably increase the serum 25(OH)D concentration to the end of supplementation. Further details of the estimated parameters and confidence intervals can be found in the Supplementary Materials (Table 3).

## Discussion and conclusions

Through re-evaluation of publicly available data, we have shown how outcome-based approaches [9] may be used to retrieve additional insights useful in a PN context.

In fact, we found that baseline serum 25(OH)D concentrations could predict serum 25(OH)D concentrations after intervention. Specifically, for individuals starting with higher baseline concentrations, the intervention by the higher dose could be required in order to reach a further increment. This finding was consistently observed in both analysed datasets. In fact, in the first dataset we observed that an individual having a baseline concentration of 40.5 ng/ml needs to receive the higher supplementation of 7000 IU/day to considerably increase the serum 25(OH)D concentration. In the second dataset we observed that an individual having a baseline concentration of 20 ng/ml needs to receive the higher supplementation of 3200 IU/day to considerably increase the serum 25(OH)D concentration. These findings are also consistent with a general S-shaped intake-response curve for a nutrient (compare for instance Heaney [10], Fig. 1). Concomitantly, these results are obtained under the assumption that a higher serum 25(OH)D concentration is always beneficial, i.e., a higher value is more advantageous than a lower value. In this form, these findings may help to understand the mechanism and the efficacy of a supplementation. In practice, compliance with the Recommended Dietary Allowances will also play a role in deciding whether supplementation is necessary at all.

Moreover, we have observed an inverse association between serum 25(OH)D concentration and body weight. While the presented statistical analyses confirm the inverse association between serum 25(OH)D concentration levels and anthropometric measures of body size (such as body weight), which is already documented in the literature [15], the mixed age population limits generalizability of these results and further studies would be necessary. In the further dataset we have observed a gender effect, but again of limited generalizability due to the unbalanced design by gender of the second dataset and the very small female population.

Also, body weight could be used as a predictor for the net gain of choosing one intervention over the other and further studies could add insights if a volumetric dilution or sequestration were to blunt the effect of higher doses.

The data under analysis were not collected to answer the present research question; this resulting secondary analysis ended with a relatively low sample size in the first subgroup of individuals with baseline serum concentrations below 30 ng/ml, whereby findings may be sensitive to few individuals and lack generalizability. Further studies are needed, possibly with larger sample sizes.

The study participants underwent “classical”, standardized interventions. The manuscript does not deal with a personalized intervention, rather it deals with estimation of individualized responses to classical interventions in order to develop criteria for a potential, future personalized intervention. The final aim will be to effectively tailor supplementation strategies by incorporating individual level predictor information. But prior to adopting any concentration threshold in practice, it is crucial to evaluate its value in improving the desired outcomes.

Concerning the statistical methods, while a subgroup analysis shows some shortcomings focusing on mean outcomes and using pre-defined cutting values, the new parametric and semiparametric models do not show these shortcomings, estimating truly individualized intervention effects. Showing the results by a subgroup analysis and by the prediction models allows to better appreciate the added value of using the newer and more targeted approaches.

It would be meaningful to include a greater number of predictors, beyond baseline concentration, age or body weight. However, as the number of predictors increases, the data becomes sparser. One well known related issue is the so-called curse of dimensionality. As a result, it will take a much larger number of individuals to draw conclusions [7]. Moreover, an increased number of predictors, also with respect to the sample size, could result in overfitting in the prediction modeling. For this scenario, shrinkage strategies could be adopted in the semi-parametric model, in order to reduce the problem of overfitting [17].

Conversely, we want to underline that a covariate known as highly significant in regression modeling and therefore well-known in the literature to be related with the outcome of interest, may not result in large improvements in prediction and therefore may not be a good predictor candidate [7].

The presented parametric and semi-parametric prediction models as generalized regression models are methods of supervised machine learning. As is well known in the machine learning literature, it would be important to gauge the predictive capability in a test dataset, i.e., through evaluation of the “learned model” by individuals who were not part of the training dataset used for fitting the original model. This can be done by employing and implementing so-called cross-validation techniques. This remains in this case open and could be further explored. Closely related to the predictive capability of the presented models, their generalization performance should be again evaluated, using a validation dataset [17].

In principle, similar results can be derived for other micronutrients and for other outcomes of interest. In fact, the proposed approaches can be directly applied to data from further clinical trials, also without the need of planning new trials. Moreover, these approaches can also be applied data comparing more than two dietary interventions. Finally, these approaches can be applied to RCTs under a crossover design.

## Supplementary information

Supplementary Materials

## Data Availability

Code and output described in the article are made publicly and freely available at 10.5281/zenodo.5546570 . Data described in the article are available at https://journals.plos.org/plosone/article?id=10.1371/journal.pone.0117123. and at https://journals.plos.org/plosone/article?id=10.1371/journal.pone.0071042.
