# Supplementary material for: The role of baseline serum 25(OH)D concentration for a potential personalized vitamin D supplementation
Source: Eur J Clin Nutr. 2022 May 23;76(11):1624–9. doi: 10.1038/s41430-022-01159-6 (PMC9630113; doi:10.1038/s41430-022-01159-6)
Supplement: Supplementary file 1 — Supplementary Materials [file 41430_2022_1159_MOESM1_ESM.docx]

**Supplementary Materials**

**Table 1. Serum 25(OH)D concentration at the begin and the end of intervention for the two supplementation doses, overall and in subgroups**

|  | **Baseline** | | **12 weeks** | |
| --- | --- | --- | --- | --- |
|  | **4000 IU** | **7000 IU** | **4000 IU** | **7000 IU** |
| Total N | 30 | 30 | 30 | 29 |
| 25(OH)D concentration, overall | 36.5 ± 9.3 | 34.5 ± 9.5 | 54.8 ± 13.0 | 56.5 ± 22.4 |
| Baseline 25(OH)D$\leq$30 ng/ml  N in group S1 | 24.0 ± 4.0  7 | 24.3 ± 4.2  9 | 45.3 ± 17.8  7 | 42.8 ± 20.4  9 |
| Baseline 25(OH)D>30 ng/ml  N in group S2 | 40.3 ± 6.6  23 | 39.0 ± 7.5  21 | 57.7 ± 9.9  23 | 62.7 ± 20.8  20 |

All concentration values given as mean ± SD and in ng/ml; *S1: subgroup of participants who started with insufficient (<= 30 ng/ml) serum 25(OH)D concentration at baseline; **S2: subgroup of participants who started with sufficient (> 30 ng/ml) serum 25(OH)D concentration at baseline

**Table 2. Estimated net gain in the 25(OH)D concentration (in ng/ml) for preferring one dose over the other with marginal confidence intervals for different baseline serum 25(OH)D concentration values in the first dataset, by Steenhoff et al.**

| **Baseline 25(OH)D** | **Estimated net gain** | **2.5 %** | **97.5 %** |
| --- | --- | --- | --- |
| 15.30 (Min) | -10.1833 | -27.4376 | 7.0711 |
| 28.55 (1^st^ Qu.) | -0.30869 | -9.52578 | 8.9084 |
| 33.60 (Median) | 3.4548 | -4.2422 | 11.152 |
| 35.54 (Mean) | 4.9006 | -2.6818 | 12.483 |
| 41.85 (3^rd^ Qu.) | 9.60317 | 0.49319 | 18.713 |
| 55.40 (Max) | 19.7013 | 2.4096 | 36.993 |

**Table 3. Estimated net gain in the 25(OH)D concentration (in ng/ml) for preferring one dose over the other with marginal confidence intervals for different baseline serum 25(OH)D concentration values in the second, independent dataset, by Carlberg et al.**

| **Baseline 25(OH)D** | **Estimated net gain** | **2.5 %** | **97.5 %** |
| --- | --- | --- | --- |
| 14.36 (Min) | 2.39 | -8.48 | 13.26 |
| 21.6 (1^st^ Qu.) | 6.30 | 2.03 | 10.58 |
| 24.04 (Median) | 7.62 | 3.89 | 11.36 |
| 23.376 (Mean) | 7.26 | 3.57 | 10.96 |
| 25.64 (3^rd^ Qu.) | 8.49 | 4.11 | 12.86 |
| 29.28 (Max) | 10.46 | 3.07 | 17.85 |
